# Feasibility and effectiveness of WhatsApp online group on breastfeeding by peer counsellors: a single-blinded, open-label pilot randomized controlled study

**DOI:** 10.1186/s13006-022-00535-z

**Published:** 2022-12-22

**Authors:** Heidi S. L. Fan, M. Y. Ho, Rachel W. T. Ko, Jojo Y. Y. Kwok, P. H. Chau, Janet Y. H. Wong, M. P. Wang, Kris Y. W. Lok

**Affiliations:** School of Nursing, Li Ka Shing Faculty of Medicine, The University of Hong Kong, 5/F Academic Building, 3 Sassoon Road, Pokfulam, Hong Kong

## Abstract

**Introduction:**

With mobile technologies becoming more advanced and accessible, mobile health (mHealth) has been incorporated in delivering timely and convenient breastfeeding support. However, its feasibility and potential efficacy remain to be examined. Therefore, the primary objective of this study is to assess the feasibility and acceptability of an online instant messaging peer support group for breastfeeding. The secondary objective is to evaluate the effect of the intervention on breastfeeding outcomes.

**Methods:**

A pilot randomized controlled trial was conducted. A total of 33 primiparous women were recruited in the antenatal clinic at a public hospital in Hong Kong between March and April 2021. They were randomized to receive either standard care (*n* = 18) or standard care and receive peer-group support in an online instant messaging app (*n* = 15). Participants received telephone follow-up for up to six months postpartum or until they stopped breastfeeding. After completing the study, six participants in the intervention group were interviewed to understand their perceptions of the intervention.

**Results:**

This pilot study shows that online messaging peer support group is feasible and acceptable to women. In total, 54.4% of the eligible women agreed to participate, and 97.0% completed the follow-up. Participants perceived that providing peer support through instant messaging app is appropriate. It serves as a channel for the participants to ask questions and obtain information. Furthermore, meetings of the peer supporters and group members can be held to enhance the effectiveness of the intervention. In addition, no significant differences were found in any and exclusive breastfeeding rates, breastfeeding attitude, and breastfeeding self-efficacy between the two groups.

**Conclusions:**

This study shows that online messaging peer support group is feasible and acceptable. A full-scale study should be conducted to understand the effect of the online instant messaging peer support group on breastfeeding outcomes.

**Trial Registration:**

The study protocol is registered on Clinicaltrial.gov (NCT04826796) on 1 April 2021

## Introduction

### Background

Breastfeeding is beneficial to both maternal and infant health [1]. It is recommended that infants should be exclusively breastfed for the first six months of their lives. World Health Organization (WHO) has encouraged the introduction of breastfeeding peer support to promote exclusive breastfeeding rates and support the needs of breastfeeding women. In Hong Kong, breastfeeding support such as breastfeeding classes, professional support, and telephone support hotline, is provided during antenatal and postnatal care in public and private settings.

Despite the growing effort in promoting breastfeeding in Hong Kong, the breastfeeding rate at 6 months has decreased from 46.5% in 2018 to 43.1% in 2020 [2]. Similarly, the exclusive breastfeeding rate at 6 months has dropped from 26.3% in 2018 to 22.2% in 2020 in Hong Kong [2]. In comparison to studies conducted during the pandemic in other countries, similar decline in any and exclusive breastfeeding rates was observed [3, 4]. It is suggested that the reduced access to breastfeeding support during the pandemic due to social-distancing measures may have contributed to this decrease.

The uncertainty of the pandemic and its related restrictions further highlights the need for alternatives to delivering breastfeeding support to women. Mobile Health (mHealth) is a popular option, which is the delivery of health care and health promotion using mobile electronic devices [5]. With the widespread use of mobile technologies and the need for timely and easily-accessible support, mHealth has been adopted as an acceptable and affordable tool in different fields, including promotion of physical activities [6], healthy diets [7], smoking cessation [8] and reducing alcohol use [9]. A meta-analysis has also found that mHealth significantly improved exclusive breastfeeding initiation, breastfeeding attitude, and knowledge [10].

According to Bandura’s Social Learning Theory, people learn through observing and imitating others’ behaviours [11]. Peer support is defined as the provision of support by an individual who had experiential knowledge of a behaviour and shared similar characteristics as the target population [12]. Peer counsellors provide role models for women to sustain breastfeeding [13]. Previous studies show that peer support interventions can significantly prolong breastfeeding duration [14]. In a previous study on home-based breastfeeding peer support in Hong Kong, participants have suggested that WhatsApp messaging groups could be a more convenient alternative for them to obtain breastfeeding peer support [15]. While there are existing professional and peer support delivered via telephone hotlines in Hong Kong [16], the feasibility and effectiveness of breastfeeding peer support delivered using an online text messaging group are yet to be examined. In addition, studies into the delivery of breastfeeding peer support using online text messaging groups remains limited. This pilot randomized controlled study (RCT) is thus proposed to evaluate the feasibility, acceptability, and potential efficacy of the intervention and study design.

### Objectives

The primary objective was to assess the feasibility and acceptability of the online instant messaging peer support group. The secondary objective of this study was to evaluate the effect of the intervention on breastfeeding outcomes.

## Methods

### Trial design

A two-armed assessor-blinded pilot RCT was conducted. The details of the pilot RCT are published elsewhere [17]. A trained research assistant recruited pregnant women at the antenatal clinic in a public hospital in Hong Kong between 5 March and 9 April 2021. They were randomized to either control or intervention group at a 1:1 ratio.

### Participants

Women were eligible to participate in the study if they (1) were primiparous, (2) intended to breastfeed, (3) had a singleton pregnancy, (4) had term infant (37–42 weeks gestational), (5) were Cantonese-speakers, (6) were Hong Kong residents and (7) had no serious medical or obstetrical complications. Women were excluded from the study if their infant (1) had an Apgar score below 8 at five minutes, (2) had a birth weight of < 2500 g, (3) had any severe medical conditions or congenital malformations, (4) was placed in the special care baby unit for more than 48 hours after birth, and (5) was placed in the neonatal intensive care unit at any time after birth.

Due to COVID-19 pandemic-related restrictions, participants recruitment was conducted in the antenatal clinic instead of the postnatal unit. Participants were screened for eligibility at the recruitment. Medical records were retrieved after the participants gave birth to their infants. Then, participants were screened for eligibility again, and they were excluded if they did not meet the inclusion criteria.

### Randomization, procedures, and intervention

Before the participant recruitment, an independent researcher who did not participate in the participant recruitment or data collection generated the allocation sequence using Stata 16 (Stata Corp., College Station, TX, USA). The sequence was concealed in a password-encoded excel file, which is not accessible to the research assistant who recruited the participants and performed data collection. After the participant recruitment and collection of the baseline data, a second research assistant accessed the sequence and notified the participants of their group assignment. Given the nature of the intervention, a single-blinded, open-label design was used. The research assistant who recruited the participants and conducted the telephone follow-up were blinded to group allocation.

Participants completed a self-administered questionnaire immediately after agreeing to participate in the study in the antenatal clinic. The self-administered questionnaire contained questions related to sociodemographic characteristics, family members’ infant feeding preferences, breastfeeding self-efficacy, and breastfeeding attitude.

Participants were randomized to the control group and intervention group after completing the self-administered questionnaire. The control group received standard care. The intervention group received standard care and peer support group on a popular online messaging mobile app, WhatsApp (“WhatsApp group”). Participants were informed before adding to the WhatsApp group. They were added into the WhatsApp group within two days after recruitment. Three trained peer counselors hosted the WhatsApp group. The peer supporters were women with at least 2-month breastfeeding experience and trained to provide breastfeeding peer support under the Department of Health in Hong Kong [18]. Once they joined the WhatsApp group, participants received a welcome message, introducing the peer counselors and encouraging them to ask questions and discuss breastfeeding-related issues. Peer counsellors provide the emotional, informational, and appraisal support [12] to the participants. They sent prompts asking for questions and providing breastfeeding-related information weekly for six months. In addition, they gave advice, shared their experience, and answered questions from participants when asked. Participants were also welcomed to share their experiences in the WhatsApp group.

Participants in both groups received telephone follow-up at 1, 2 and 4, and 6 months postpartum or until they stopped breastfeeding, which ever came first. The proportion of different types of infant feeding during the preceding 24 hours was asked in each follow-up. The breastfeeding self-efficacy and breastfeeding attitude were assessed in the second month postpartum. In addition, participants in the intervention group were asked about their experiences in participating in the online peer support group discussion in each telephone follow-up.

After study completion, participants in the intervention group were invited to participate in a structured interview. Participants’ perceptions of the interventions and suggestions on improving the effectiveness of the interventions were explored. The interviews were conducted either in a face-to-face interview or via zoom. The length of interviews ranged from approximately 20 to 60 minutes.

### Study outcomes

For the primary objective, the intervention’s feasibility and acceptability were assessed. To measure the feasibility, the proportion of women who agreed to participate, and completed the follow-up were examined. The acceptability of the intervention was measured by the perceived helpfulness of the intervention and the perceptions of the intervention. Participants were asked to rate the helpfulness of the intervention in each telephone follow-up, with means “not at all helpful” and 10 means “extremely helpful”. Additionally, the perceptions of the intervention were assessed in the qualitative interviews. The views and feedback of participants on the intervention were described.

For the secondary objective, the breastfeeding outcomes were assessed. The breastfeeding outcomes assessed were the proportions of any breastfeeding and exclusive breastfeeding at 1, 2, 4, and 6 months postpartum. In addition, the participants’ breastfeeding self-efficacy and breastfeeding attitude were measured at two months postpartum. According to WHO definitions [19], any breastfeeding is defined as the infant receiving any breast milk, regardless of the mode of breastfeeding. In addition, exclusive breastfeeding is defined as feeding only breast milk and no other liquids or solids, except for vitamin and mineral supplements or medicines [19].

Breastfeeding self-efficacy was measured using the Breastfeeding Self-Efficacy Scale-Short Form (BSES-SF) [20]. BSES-SF comprises 14 items and applied a five-point Likert scale from 1 to 5. The total score ranges from 14 to 70, with higher scores indicating a higher level of breastfeeding self-efficacy. A translated and validated Chinese version of the BSES-SF was used in this study [21]. Furthermore, the breastfeeding attitude of the participants was measured using the Iowa Infant Feeding Attitude Scale [22]. IIFAS consists of 17 items with a five-point Likert scale. On the scale, “1” means “strong disagreement” and “5” means “strong agreement”. The total score ranges from 17 to 85, with higher score indicating a more favorable attitude towards breastfeeding. The Chinese version of the IIFAS was used in this study [23]. The BSES-SF and IIFAS were administered to the participants at baseline and two months postpartum. Only participants who were still breastfeeding completed the BSES-SF and IIFAS at two months postpartum.

### Sample size

The sample size of the trial is 40 participants, with 20 participants in each arm. This is the recommended sample size for feasibility and pilot study that estimates the recruitment rate retention and identifies unanticipated issues of the trial [24]. However, the proposed sample size was not reached as 10 participants have later met the exclusion criteria.

### Data analysis

The intervention and control groups’ baseline characteristics were compared using Fisher’s exact tests and independent samples t-test. Cohen’s d effect sizes *(d)* were calculated and considered as trivial (< 0.2), small (≥0.2 and < 0.5), moderate (≥0.5 and < 0.8) or large (> 0.8) [25]. A professional transcriptionist transcribed the interviews into Cantonese and translated the transcripts from traditional Chinese to English. The recordings were reviewed several times to ensure that the data were interpreted accurately. Content analysis was used to analyze the transcripts. The core consistencies and meanings in the qualitative data were found [26]. The data analysis was conducted manually and assisted with a word processing program.

Furthermore, the proportion of any and exclusive breastfeeding at 1, 2, 4, and 6 months were compared using Fisher’s exact tests. In addition, independent t-tests were used to compare the BSES-SF and IIFAS at baseline and 2 months postpartum between the two groups. Intention-to-treat analysis was conducted. Participants who were lost to follow-up was treated as not breastfeeding. All quantitative data were analyzed using STATA Version 16. We used a 95% confidence interval and a 5% significance level in all statistical tests.

### Trial registration and ethical approval

The study protocol is registered on Clinicaltrial.gov (NCT04826796) on 1 April 2021. This study was reviewed and approved by the Institutional Review Board of the University of Hong Kong/ Hospital Authority Hong Kong West Cluster (Reference: UW 21–039) on 26 January 2021 and was conducted in accordance with the Declaration of Helsinki and its later amendments. All participants have provided written informed consent to participate.

## Results

**Fig. 1:**
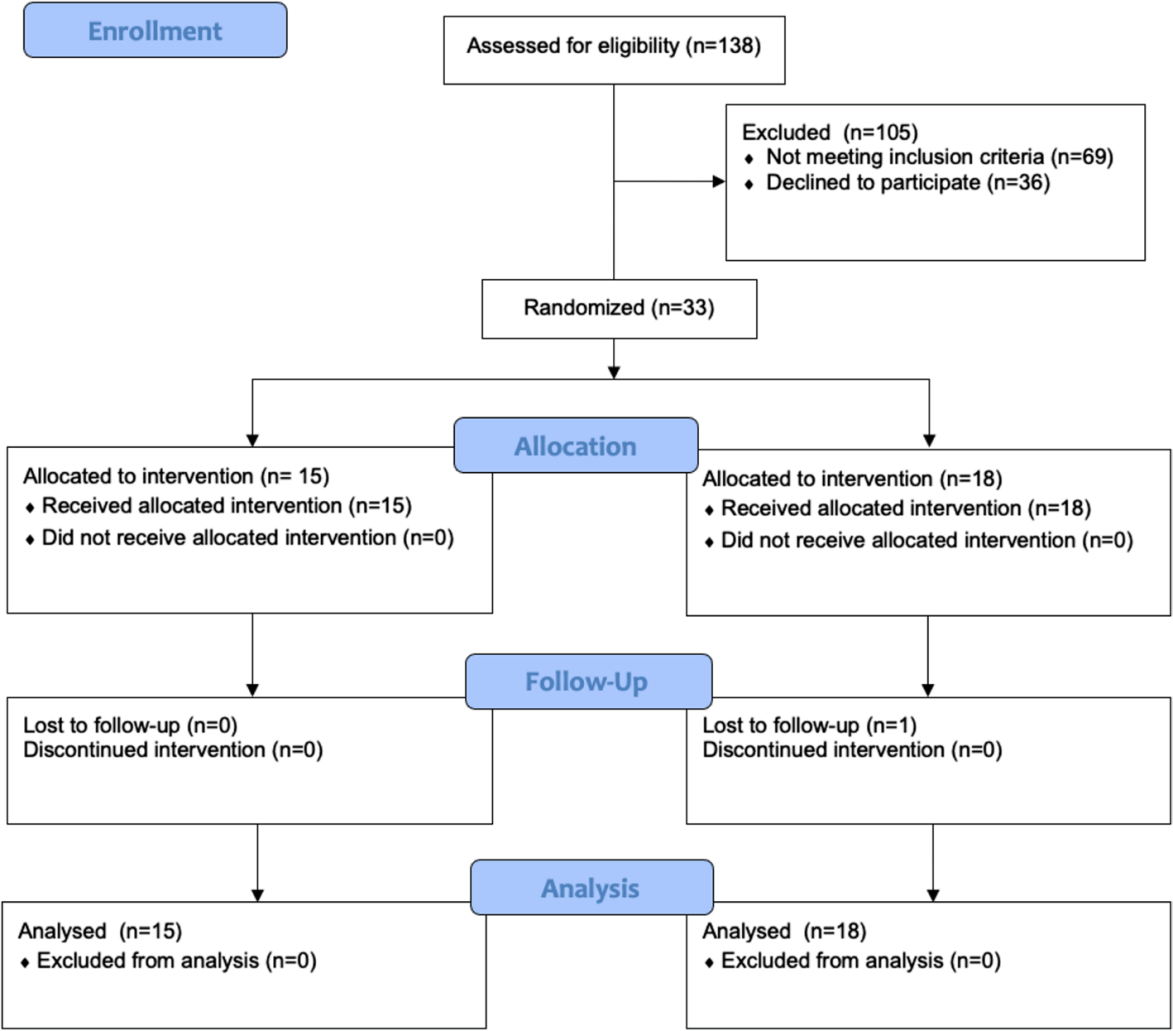
Flow diagram of participant recruitment and follow-up

Figure 1 shows the flow diagram of participant recruitment. A total of 138 pregnant women were screened for eligibility, 69 of whom were ineligible, and another 36 declined to participate. Therefore, a total of 33 participants were included in the analysis. There are 15 participants randomized into the intervention and 18 participants into the control group. One participant in the control group did not complete the telephone follow-up at six months postpartum. The comparisons of the baseline characteristics of the participants are presented in Table 1. No statistical differences were found between the two groups.

**Table 1:** Demographic characteristics of study participants in intervention and control groups

	Total *N* = 33 (%)	Intervention *N* = 15 (%)	Control *N* = 18 (%)	*p*-value
Maternal age (year), Mean (SD)	33.0 (4.4)	32.1 (4.4)	33.7 (4.4)	.290
Maternal education				.418
< University degree	8 (24.2)	5 (33.3)	3 (16.7)	
University Degree or above	25 (75.8)	10 (66.7)	15 (83.3)	
Monthly family income (HKD)^a^				.850
< $20,000	3 (9.1)	1 (6.7)	2 (11.1)	
$20,000–$39,999	5 (15.2)	3 (20.0)	2 (11.1)	
≥ $40,000	25 (75.8)	11 (73.3)	14 (77.8)	
Born in Hong Kong				.239
No	9 (27.3)	6 (40.0)	3 (16.7)	
Yes	24 (72.7)	9 (60.0)	15 (83.3)	
Intention to return to work postpartum				.665
No	6 (18.2)	2 (13.3)	4 (22.2)	
Yes	27 (81.8)	13 (86.7)	14 (77.8)	
Intention to exclusively breastfeed				.296
No	18 (54.6)	10 (66.7)	8 (44.4)	
Yes	15 (45.5)	5 (33.3)	10 (55.6)	
Partner’s infant feeding preference				1.000
Breastfeeding	13 (39.4)	6 (40.0)	7 (38.9)	
Infant formula & mixed feeding	19 (57.6)	9 (60.0)	10 (55.6)	
No preference	1 (3.0)	0 (0.0)	1 (5.6)	
Attended childbirth class (es)				.732
No	17 (51.5)	7 (46.7)	10 (55.6)	
Yes	16 (48.5)	8 (53.3)	8 (44.4)	
Attended breastfeeding class (es)				.729
No	18 (54.6)	9 (60.0)	9 (50.0)	
Yes	15 (45.5)	6 (40.0)	9 (50.0)	
Mode of birth				.493
Spontaneous vaginal	17 (51.5)	10 (66.7)	7 (38.9)	
Assisted vaginal	6 (18.2)	2 (13.3)	4 (22.2)	
Planned caesarean	5 (15.2)	1 (6.7)	2 (22.2)	
Emergency caesarean	5 (15.2)	2 (13.3)	3 (16.7)	

^a^1 USD = 7.78 HK

### Feasibility and acceptability

As mentioned before, 54.4% of the eligible women agreed to participate, and 97.0% completed the follow-up. In addition, participants in the intervention group have rated the peer support group to be somewhat helpful throughout the follow-up periods, with an average score of 6.21 (SD = 1.79) at 1 month, 6.08 (SD = 1.55) at 2 months, 6.38 (SD = 1.21) at 4 months, and 6.45 (SD = 1.83) at 6 months [data not shown].

Among the 15 participants in the intervention group, 6 agreed to be interviewed. The structured interviews ask for participants’ perceptions of the intervention. In addition, they were invited to make suggestions on improving the effectiveness of the intervention.

### Perceptions of the intervention

Participants found that the mode of intervention provision is appropriate. They perceived the WhatsApp group as a tangible and emotional support channel. However, they were concerned about privacy issues and unfamiliar with other group members. In addition, participants suggested that the WhatsApp group can be more age-specific and approachable.

### Provision of peer support through WhatsApp group

All the participants found that the WhatsApp group is appropriate for providing peer support. It is convenient as they can receive instant support. In addition, participants can read the messages and participate in the conversations anytime when they are free. However, participants had varying opinions on the group size. Some participants prefer to have more mothers in the group, while others prefer fewer people.

“The best thing about the WhatsApp group is that (the response) can be speedy. You can leave a message, and others can reply to you anytime when they think it is appropriate. I think it is very convenient. I don’t need to worry about “Will I disturb others if I leave my message now?“ …. I don’t have this worry now. If it is not a WhatsApp group, e.g., calling the peer supporters, I may be worried that there will be no one listening to my phone calls at this time and will disturb others.” (Participant #4).

### A channel of tangible and emotional support

Participants used the WhatsApp group as a channel to ask questions and obtain information. Some participants considered it as a source of emotional support and reassurance. Participants found that it was helpful to know that other mothers in the group also faced the same problems and that they were not alone.

“It is quite helpful for new mothers.... I read others’ questions and comments sometimes. I will know the answers and obtain some basic knowledge. When I face the same situation, I am not nervous about it and know what to do …. (The WhatsApp group) is somewhat helpful. When you encounter problems (about infant feeding), you are very worried. As we have this WhatsApp group, someone tells you that you don’t need to be afraid, which is normal. At least I felt less nervous at that moment.” (*Participant #6*).

“I think the WhatsApp group is helpful. Otherwise, I may stop breastfeeding when my baby is not willing to be fed. .... Sometimes, I read the WhatsApp messages and the peer supporters send the information to us. The information is somewhat helpful. The advantage of the group is that I can know that others are also in a similar situation as me.” (Participant #5).

### Privacy concerns and unfamiliarity with other group members

Some participants considered themselves inactive in the group. They felt uncomfortable discussing their situations as they did not know the others. They prefer to ask family members or friends when they encounter breastfeeding problems. In addition, they were unfamiliar with other group members. They would be afraid that asking questions in the group will bother other group members and the volunteers.

“I felt embarrassed (to ask questions). It is because I don’t know the people in the group. Therefore, I did not ask them questions. …. I cannot say that I don’t trust them. However, I prefer to ask someone I know, the people in my social network.” (Participant #5).

“I think it is because of the COVID-19 pandemic. I may not know who are included in the WhatsApp group. When there are lots of strangers, this hinders me from asking questions. I think, “Can I ask this question? Would others mind that I ask this question?”” (Participant #3).

“I have asked questions only once or twice. My questions are not big problems. Therefore, I think (the WhatsApp group) is OK. … I did not ask questions frequently as I was worried that I would bother others. I was not willing to ask questions. …. In addition, there are not many interactions in the group. I guess other group members are as busy as me. Therefore, it will not be the same as the WhatsApp groups with the family members. It is rare for all of us to keep chatting. “(Participant #6).

### Suggestions for improvements: the formation of an age-specific and approachable group

Some participants reflected that the group included mothers who gave birth at different times. Therefore, breastfeeding problems that the mothers encountered may be different. They suggested that the group could consist of mothers with infants of similar ages. In addition, to improve the sense of familiarity within the group and establish an approachable atmosphere, some participants suggested that every group member can introduce themselves when they join the group, or that they can have a meeting before joining the group.

“When there are different people asking questions, the volunteers also need to answer different questions, and it can be unclear. In addition, it keeps on having new mothers join the group. Mothers may have different needs when their infants were at different ages, e.g., a mother just gave birth, and another mother was already at 7 months postpartum. The problems that they face will be very different. The group can be separated into two groups. …. It would be better if we met once. It is because I know what you look like, and I know the background of the peer supporters. I would be more willing to ask questions or rely on the peer supporters.” (Participant #3).

### Breastfeeding outcomes

**Fig. 2:**
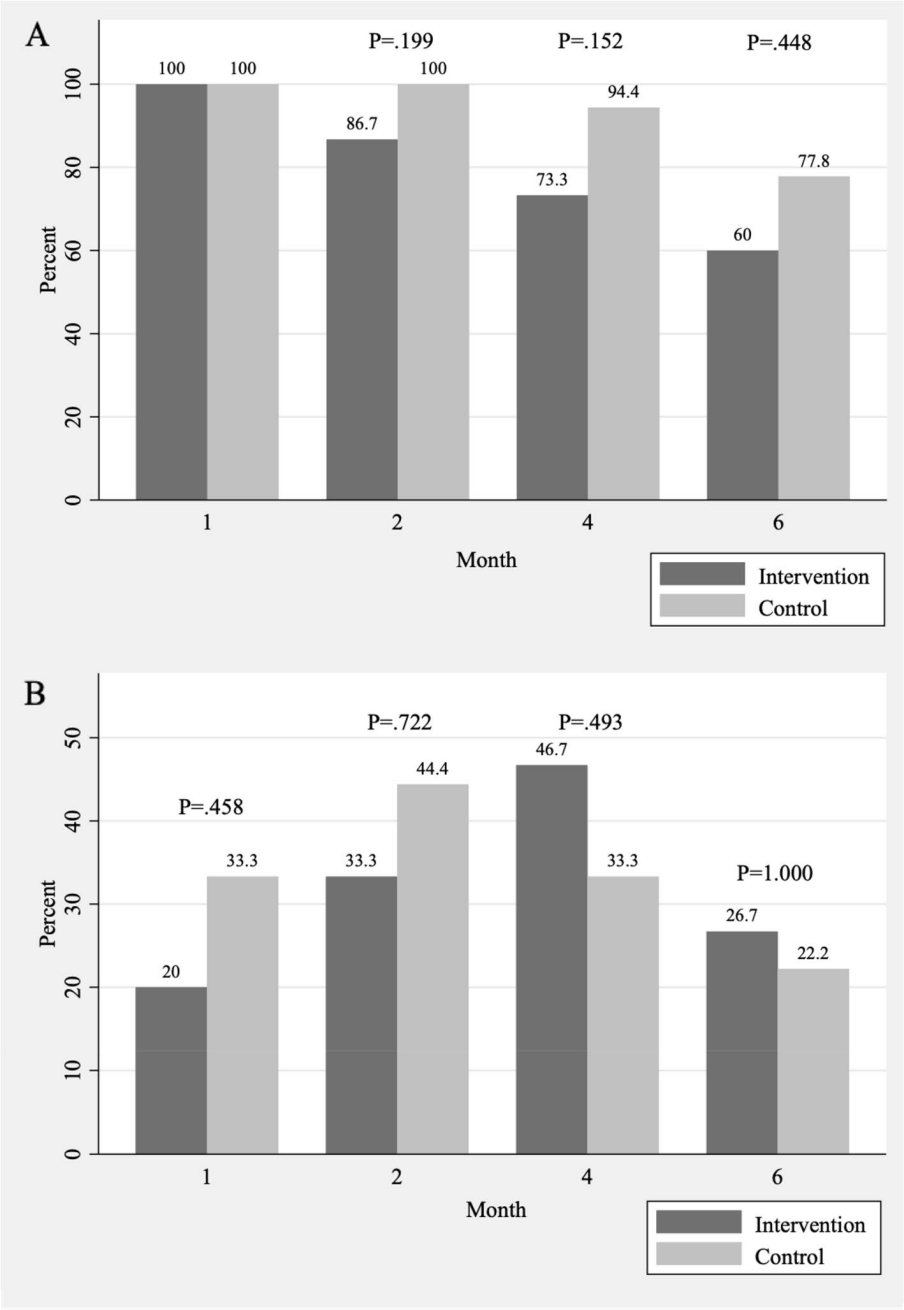
Proportion of (**A**) any breastfeeding and (**B**) exclusive breastfeeding by treatment group over the first six months postpartum

Figure 2 displays the proportion of participants who were any and exclusive breastfeeding at the four study timepoints. All the participants continued to breastfeed at one month postpartum. A high proportion of participants continued to breastfeed at 6 months postpartum, with 60% in the intervention group and 77.8% in the control group, yet such difference did not reach statistical significance (*P* = .448). As for exclusively breastfeed, when compared with the control group, participants in the intervention group were not more likely to at 1 month (33.3% vs. 20%; *P* = .458), 2 months (44.4% vs. 33.3%; *P* = .722), 4 months (33.3% vs. 46.7%; *P* = .493) and 6 months (22.2% vs. 26.7%; *P* = 1.000) postpartum.

Table 2 presents the BSES-SF scores and IIFAS scores at baseline and 2-month follow-up by treatment group. The total sample’s overall mean BSES-SF scores at baseline were 39.27 (standard deviation [SD] = 9.22). The BSES-SF scores increased at 2 months postpartum, whereas no statistical difference was shown between the control and intervention groups (*d* = − 0.30; *P* = .423). Similarly, the IIFAS scores increased at 2 months postpartum in the control (mean [M] = 62.89, SD = 5.58) and intervention (M = 61.23; SD = 6.61) group. There were no significant differences between groups at two months postpartum (*d* = − 0.28; *P* = .456).

**Table 2:** Breastfeeding self-efficacy and attitude at study entry and 2-month postpartum in intervention and control group

		Total Mean (SD)	Intervention Mean (SD)	Control Mean (SD)	Statistics	Cohen’s D effect Size	*p*-value
BFSES	Baseline	39.27 (9.22)	37.13 (8.56)	41.06 (9.61)	t(31) = −1.23	−0.43	.230
	2-month	46.87 (10.39)	45.08 (9.78)	48.17 (10.90)	t(29) = −0.81	−0.30	.423
IIFAS	Baseline	60.03 (5.34)	60.13 (5.99)	59.94 (4.90)	t(31) = 0.10	0.03	.921
	2-month	62.19 (5.99)	61.23 (6.61)	62.89 (5.58)	t(29) = −0.76	−0.28	.456

## Discussion

This pilot study shows that online messaging peer support group is feasible and acceptable to the participants. Participants perceived that providing peer support through instant messaging app is appropriate. It serves as a channel for the participants to ask questions and obtain information. However, not knowing the other group members make them reluctant to ask questions or have discussions in the group. Therefore, prior meetings of the peer supporters and group members with similar infant ages can be strategies to enhance the effectiveness of the intervention in a larger RCT design. With respect to potential efficacy of the intervention, when compared with the control arm, the intervention arm had no significant effect on the rate of any and exclusive breastfeeding at 1, 2, 4, and 6 months postpartum. However, this study was not powered to detect significant differences between groups and the study’s purpose was to identify its feasibility and acceptability, this finding might be indicative of efficacy but is not conclusive, warranting further investigation.

A meta-analysis shows that peer support effectively increases exclusive breastfeeding at 3 months postpartum [27] and the majority of the interventions in the existing studies were home-based visits or using telephone follow-up [27]. Studies show that home-based peer supports effectively improve breastfeeding outcomes [28, 29], while telephone peer support shows inconsistent effects on breastfeeding outcomes [30–34]. The mHealth interventions used in the existing studies were breastfeeding peer support groups on Facebook, forums in parenting websites, or unspecified social media [35]. To our knowledge, this is the first study to understand the effect of instant messaging groups on breastfeeding duration. Support provided through an instant messaging smartphone app has been demonstrated to be effective in other health promotion activities [36, 37]. This study has shown that providing peer support through instant messaging app is feasible and acceptable to breastfeeding women. The women recognized the benefits of receiving instant responses from peer supporters. Furthermore, this study has provided directions on enhancing the peer support through online instant messengers. Although the interventions were provided through WhatsApp, gaining familiarity with the peer supporters and other group members were one of the essential factors for the success of the interventions.

As this study is a pilot study, only a small sample size was used. This may be one of the possible reasons that online messaging peer support group has no significant effect on breastfeeding duration. The provision of peer support through instant messaging app is effective when in-person support is not feasible, such as during the COVID-19 pandemic. A high proportion of women have reported that they did not receive adequate breastfeeding support was the primary reason for breastfeeding cessation during the COVID-19 pandemic [38]. Therefore, there is an urgency to develop effective virtual breastfeeding support [39], especially for women who were unable to receive in-person support and help prepare for a future pandemic outbreak. Therefore, a full-scale study with an intervention that was modified based on the outcomes of this study would be needed to have a holistic understanding of the effect of the provision of peer support through an online messaging mobile app on breastfeeding outcomes.

## Limitations

This study has several limitations. First, a small sample of primiparous women in Hong Kong with the intention to breastfeed was recruited in this study. Therefore, the results may not be generalizable to multiparous women. In addition, recall bias may occur as the breastfeeding outcomes were self-reported by the participants. However, we have used the WHO recommended 24-hour recall of infant feeding practices [19] to minimize the recall bias. Furthermore, we did not measure other breastfeeding support that the participants received. Participants in the control group may receive other breastfeeding supports, which may reduce the differences in the breastfeeding outcomes between the intervention and control groups.

## Conclusion

This study demonstrates that online messaging peer support group is feasible and acceptable to women. In terms of potential efficacy of the intervention, it did not show significant differences for breastfeeding outcomes when compared to usual care, a finding that warrants further study. Modifications to the intervention, such as prior meetings of the peer supporters and group members with similar infant ages maybe more appropriate in a larger, adequately powered trial. A full-scale study should be conducted to understand the effect of the online instant messaging peer support group on breastfeeding outcomes.

## Data Availability

The dataset used and/or analysed during the current study are available from the corresponding author on reasonable request.

## Abbreviations

BSES-SF: Breastfeeding Self-Efficacy Scale-Short Form
mHealth: Mobile Health
RCT: Randomized Controlled Study
WHO: World Health Organization
